# Sexual dimorphism of early transcriptional reprogramming in degenerating peripheral nerves

**DOI:** 10.3389/fnmol.2022.1029278

**Published:** 2022-10-27

**Authors:** Andrei V. Chernov, Veronica I. Shubayev

**Affiliations:** ^1^Department of Anesthesiology, University of California, San Diego, San Diego, CA, United States; ^2^VA San Diego Healthcare System, San Diego, CA, United States

**Keywords:** *Mus musculus*, sexual dimorphism, peripheral nerve injury, axotomy, RNA-seq, Wallerian degeneration

## Abstract

Sexual dimorphism is a powerful yet understudied factor that influences the timing and efficiency of gene regulation in axonal injury and repair processes in the peripheral nervous system. Here, we identified common and distinct biological processes in female and male degenerating (distal) nerve stumps based on a snapshot of transcriptional reprogramming 24 h after axotomy reflecting the onset of early phase Wallerian degeneration (WD). Females exhibited transcriptional downregulation of a larger number of genes than males. RhoGDI, ERBB, and ERK5 signaling pathways increased activity in both sexes. Males upregulated genes and canonical pathways that exhibited robust baseline expression in females in both axotomized and sham nerves, including signaling pathways controlled by neuregulin and nerve growth factors. Cholesterol biosynthesis, reelin signaling, and synaptogenesis signaling pathways were downregulated in females. Signaling by Rho Family GTPases, cAMP-mediated signaling, and sulfated glycosaminoglycan biosynthesis were downregulated in both sexes. Estrogens potentially influenced sex-dependent injury response due to distinct regulation of estrogen receptor expression. A crosstalk of cytokines and growth hormones could promote sexually dimorphic transcriptional responses. We highlighted prospective regulatory activities due to protein phosphorylation, extracellular proteolysis, sex chromosome-specific expression, major urinary proteins (MUPs), and genes involved in thyroid hormone metabolism. Combined with our earlier findings in the corresponding dorsal root ganglia (DRG) and regenerating (proximal) nerve stumps, sex-specific and universal early phase molecular triggers of WD enrich our knowledge of transcriptional regulation in peripheral nerve injury and repair.

## Introduction

Successful regeneration of a severed peripheral nerve relies on a well-coordinated multicellular distal degeneration process first described by Waller (1850). Significant insight into the cellular and molecular processes of Wallerian degeneration (WD) was obtained using rodent models of the peripheral nerve transection (Fawcett and Keynes, 1990; Stoll et al., 2002; Vargas and Barres, 2007). A high-efficiency acute-phase (within 24 h) peripheral WD process in the distal to transection segment, disconnected from neuronal soma, determines functional repair of the nerve (Gordon et al., 2003; McDonald et al., 2006; Wood et al., 2011; Zochodne, 2012). These prior acute-phase peripheral WD studies were done using rodents of one sex.

Sex-dependent response to peripheral nerve injury in rodents (Sorge et al., 2015; Chernov et al., 2020; Yu et al., 2020; Chernov and Shubayev, 2021, 2022) is thought to relate to sex differences in prevalence, incidence, mechanisms, or clinical presentation of peripheral neuropathies (Unruh, 1996; Greenspan et al., 2007; Fillingim et al., 2009; Mogil, 2012; Sorge and Totsch, 2017; Boerner et al., 2018). The transcriptional landscape of peripheral nerve in control human subjects and patients with radiculopathy assessed by RNA-sequencing (RNA-seq) analyses reveal sexual dimorphism in metabolic, neuroendocrine, immune regulatory, and sex chromosome-related programs both at baseline and in response to peripheral nerve damage (North et al., 2019; Ray et al., 2019; Stephens et al., 2019; Chernov et al., 2020; Mecklenburg et al., 2020; Paige et al., 2020; Tavares-Ferreira et al., 2020; Ahlström et al., 2021). Whether sexual dimorphism in acute-phase WD exists remains not understood.

During an acute-phase (within 24 h) peripheral WD, an immediate influx of extracellular calcium activates calpain- and ubiquitin-proteasome-dependent disintegration of the axonal cytoskeleton. A dominant cell type in peripheral nerve, denervated Schwann cells initiate axonal and myelin breakdown and phagocytosis. In concert with resident macrophages, endothelial cells, and fibroblasts, Schwann cells control a time-dependent and continuous stream of hematogenous immune cells, starting with neutrophils, mast cells, monocytes, and lymphocytes (Hirata and Kawabuchi, 2002; Bauer et al., 2007; Scholz and Woolf, 2007; Barrette et al., 2008; Jessen and Arthur-Farraj, 2019). In addition, Schwann cells engage vast molecular machinery required to support their robust phenotypic changes induced by injury, including de-differentiation, accompanied by myelin protein gene silencing and glial fibrillary acidic protein (GFAP) activation, followed by neuregulin-epidermal growth factor receptor (EGFR, also named ERBB) controlled mitosis, and c-Jun-regulated redifferentiation, axonal partnership and remyelination (Clemence et al., 1989; Triolo et al., 2006). Extensive molecular signatures reveal a dynamic interplay between immune, metabolic, kinase, neurotrophic, and other gene families jointly contributing to the functional activity of many cell types in a time-dependent manner. Thus, within 24 h post-axotomy, calcium- and zinc-dependent activation of extracellular matrix (ECM) remodeling by matrix metalloproteinase (MMP) family of 24 members, which control permeability of blood-nerve and perineurial barriers, Schwann cell signaling, as well as myelin and ion channel proteolysis (Shubayev and Myers, 2002; Myers et al., 2006; Shubayev et al., 2006; Chattopadhyay et al., 2007; Kobayashi et al., 2008; Chattopadhyay and Shubayev, 2009; Liu et al., 2010, 2012; Liu and Shubayev, 2011; Kim et al., 2012; Nishihara et al., 2015; Remacle et al., 2015).

Our comparative high-depth (over 5.0E + 07 paired-end reads per sample) RNA-seq and predictive bioinformatics analyses of the early response genome-wide transcriptional changes at 24 h after sciatic nerve axotomy in male and female mice indicated immediate and sex-dependent control of the transcriptional landscape in dorsal root ganglia (DRG) (Chernov and Shubayev, 2021), regenerating (proximal) nerve segment (Chernov and Shubayev, 2022). The present study of degenerating (distal) identified new sexually dimorphic protein-coding and non-coding (nc)RNAs and respective signaling pathways specific to peripheral nerve remodeling.

## Results

### Peripheral Wallerian degeneration animal model

**FIGURE 1:**
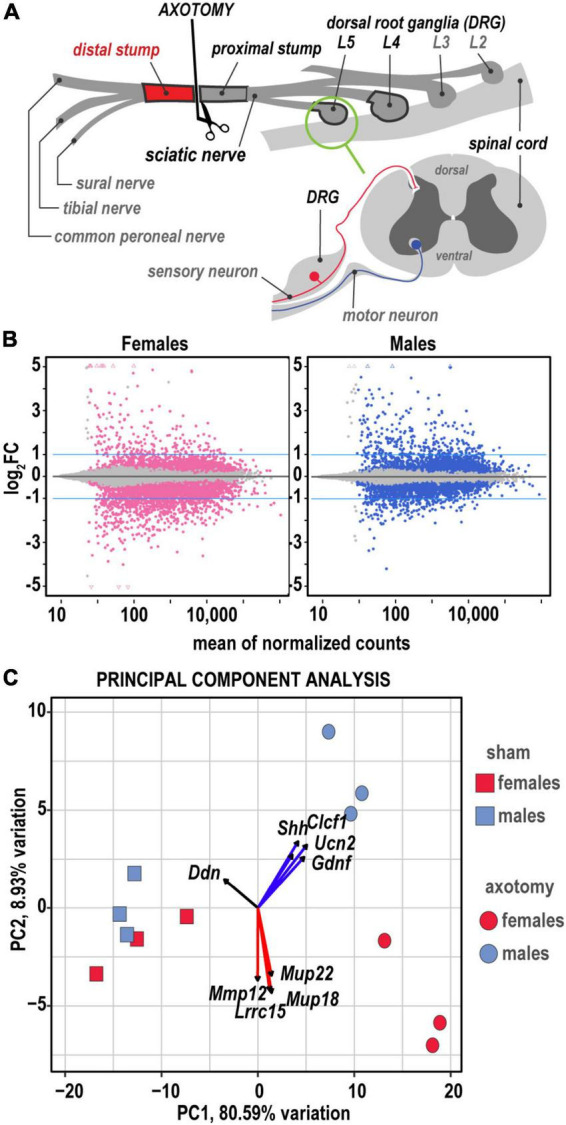
Global transcriptional profiling in distal nerve 24 h post-axotomy identified sexually dimorphic changes. **(A)** A schematic of the sciatic nerve axotomy followed by ipsilateral distal stump tissue analysis. RNA-seq of the respective lumbar (L) 4/5 DRG and proximal sciatic nerve stumps from the same animal cohorts were reported earlier (Chernov and Shubayev, 2021, 2022). **(B)** MA plots display log_2_FC and normalized counts calculated using the default *DESeq2* method. The left and right panels show up- and downregulated genes in females (red dots) and males (blue dots), respectively. Colored dots indicate DEGs with P_adj_ < 0.1. Blue lines correspond to log_2_FC thresholds above 1 or below –1. **(C)** Principal component analysis of female (red) and male (blue) groups of sham (rectangles) and axotomy (circles) samples (*n* = 3 samples/group). Red and blue arrows indicate genes with the most influence on variance in females and males, respectively.

To assess sex differences in the early phase peripheral WD transcriptional (protein-coding mRNAs and ncRNA) response, at 24 h after complete sciatic nerve axotomy or sham operation, distal nerve stumps (Figure 1A) were collected in female and male mice (*n* = 6 mice/group). To obtain the optimal amount of total RNAs for whole-genome transcriptomics analysis by high-depth RNA-seq nerve stumps from two mice were pooled in all RNA samples (*n* = 3/groups, 2 mice/RNA sample). RNA-seq of the respective lumbar (L) 4/5 DRG and proximal sciatic nerve stumps from the same animal cohorts were reported earlier (Chernov and Shubayev, 2021, 2022).

### Females exhibited transcriptional inactivation in distal nerve stumps at 24 h post-axotomy

Genes with greater than 10 transcript counts across all samples were used for normalization and identification of differentially expressed genes (DEGs) in axotomy samples relative to respective shams by DESeq2 using Wald’s test (Love et al., 2014). The standard significance criteria P_*adj*_ < 0.1 and log_2_FC > 1 were used to identify significant DEGs (Figure 1B) in this report and our prior comparative analyses of regenerating peripheral nerves (Chernov and Shubayev, 2022) and DRG (Chernov and Shubayev, 2021). Baseline gene expression [log_2_(counts)], log_2_FC, and significance scores of 1,017 DEGs identified by DESeq2 were used for predictive system analysis (Supplementary Table 1). Comparative analysis of RNA-seq and protein immunoblotting data of select genes was conducted in DRG of the same animal cohort, paired-end library preparation, and RNA-seq. A high level of correlation between mRNA and protein levels was demonstrated in post-axotomy and sham samples (Chernov and Shubayev, 2021).

**FIGURE 2:**
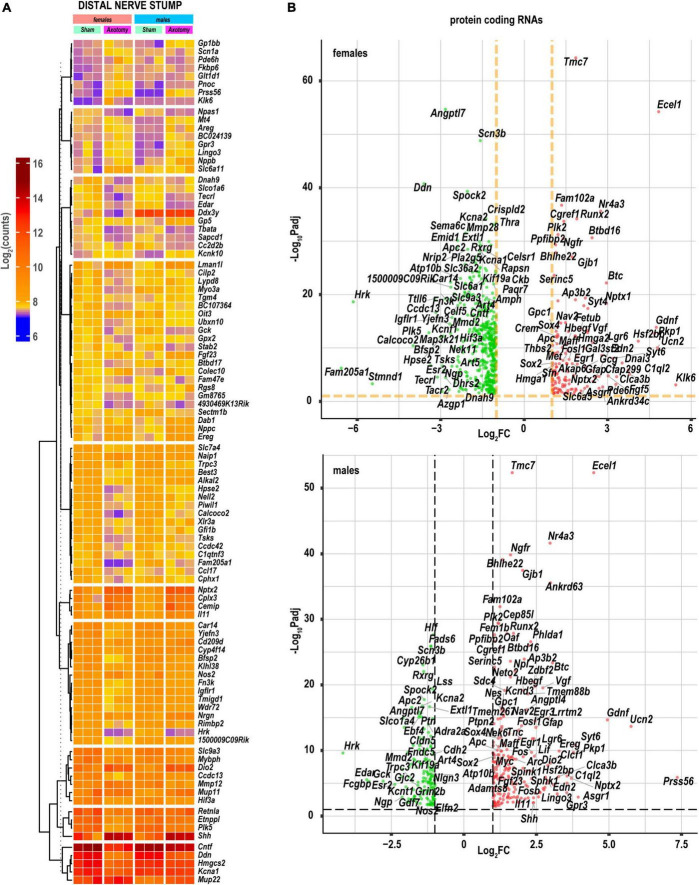
Nerve axotomy induced distinct sexually dimorphic regulation of DEGs in female and male mice. **(A)** Hierarchical clustering plot of 100 most significant upregulated DEGs [log_2_FC > 1, P_adj_ < 0.1, *n* = 6 mice/group, 2 mice/sample (pooled), 3 sample/group]. Heatmap colors correspond to log_2_(counts): blue, yellow, and red correspond to low, medium, and high gene expression, respectively. DEGs were clustered based on the Euclidean distance. **(B)** Significant DEGs in female (left panel) and male (right panel) mice. Volcano scatter plots show -log_10_P_adj_ and log_2_FC. Red and green colors indicate up- and down-regulated DEGs, respectively. Thresholds (log_2_FC > 1 or log_2_FC < –1) and –log_10_P_adj_ < 0.1 are indicated by yellow dashed lines. Selected DEG symbols are shown.

The principal component (PC) analysis (Figure 1C) identified sex-specific variance-driving DEGs related to ECM homeostasis, nerve regrowth, axonal guidance, and cytokine signaling among the most significant contributors to injury-related transcriptional changes in distal nerve stumps. We predicted that 80.6% (PC1) and 8.9% (PC2) of DEGs could contribute to variance based on axotomy and sex differences. Specifically, sonic hedgehog (*Shh*), cardiotrophin-like cytokine factor 1 (*Clcf1*), ECM-localized corticotropin-releasing factor urocortin-2 (*Ucn2*), and glia-derived neurotrophic factor (*Gdnf*), leucine-rich repeat-containing protein 15 (*Lrrc15*), and major urinary proteins *Mup18/Mup22* genes were the most significant drivers of sexually dimorphic transcriptional variance. Heatmap histograms ranked by absolute expression in three biological replicates (Figure 2A) and volcano plots (Figure 2B) display DEG’s ranked by statistical significance (P_*adj*_) and log_2_FC ranking.

### Predictive ingenuity analysis identified sexually dimorphic canonical pathways

**FIGURE 3:**
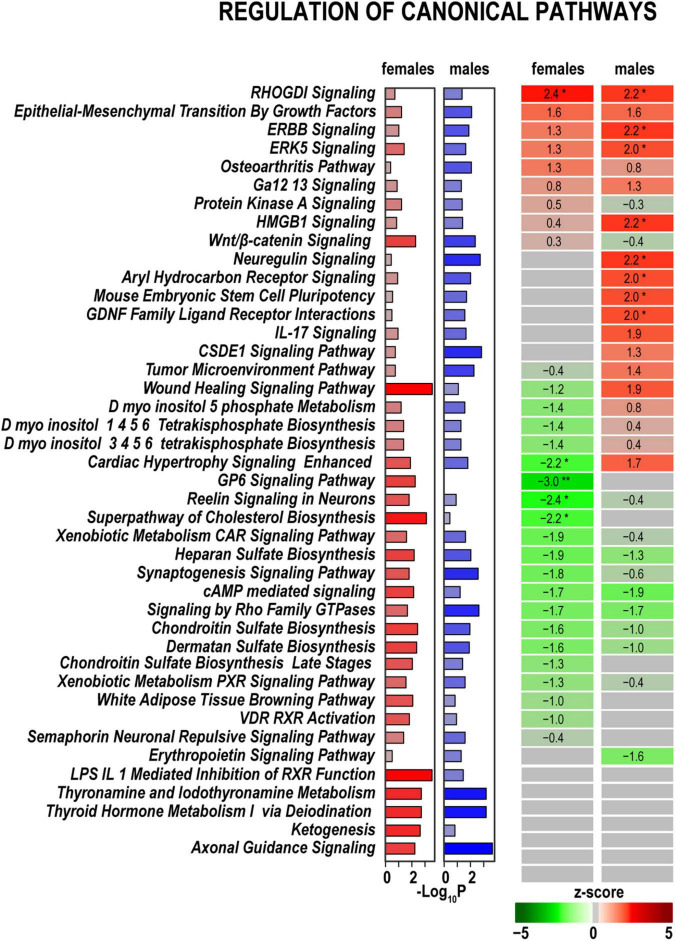
Canonical signaling pathways demonstrate distinct regulation patterns in females and males. Canonical pathways were predicted in IPA and ranked by z-scores. Positive (red) and negative (green) z-scores indicate pathway up-regulation or down-regulation, respectively, according to the color scale. Asterisks indicate significance *, z < –2.0 or z > 2.0; **, z < –3.0, or z > 3.0. The gray color indicates that the directionality of pathway regulation was not determined. Purple (females) and blue (males) horizontal bars correspond to –log_10_(P) ≥ 1.3 calculated in IPA using Fisher exact test.

**FIGURE 4:**
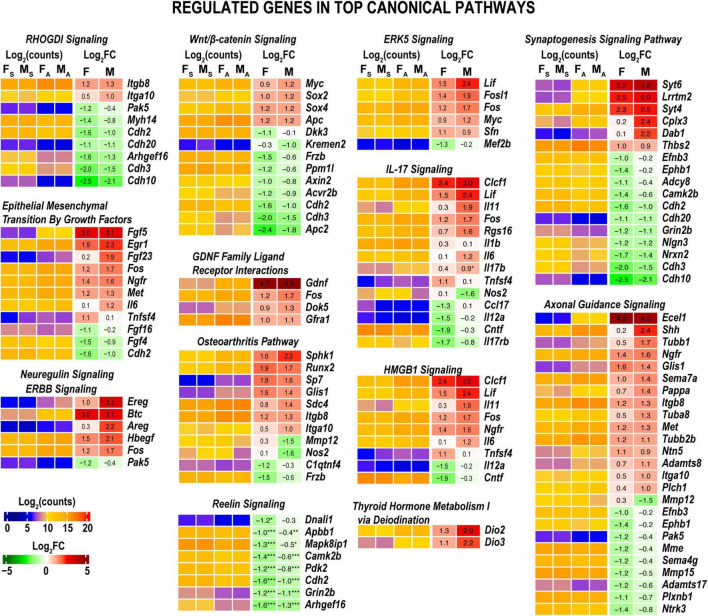
Axotomy affected major nerve injury-related canonical pathways. Histograms show DEG specific to canonical pathways highlighted in Figure 3. Heatmaps display normalized log_2_(counts) in sample groups (F_S_, female sham; M_S_, male sham; F_A_, female axotomy; M_A_, male axotomy) and log_2_FC in female (F) and male (M); [*n* = 6 mice/group, 2 mice/sample (pooled), 3 sample/group] of respective DEGs. Log_2_FC significance was determined in DESeq2 using the Wald test. DEGs were sorted by log_2_FC.

In both sexes, we identified high expression levels of genes related to signaling pathways specific to nerve injury response. Some of these pathways exhibited additional activation or inhibition at 24 h post-axotomy as outlined below. *RhoGDI, ERBB, ERK5 Signaling pathways*, and *Epithelial/Mesenchymal Transition by Growth Factors Signaling* pathway showed significant activation in both sexes (Figure 3). Compared to males, females demonstrated distinct, albeit mild, upregulation of the *Osteoarthritis Signaling, Protein Kinase A Signaling*, and *Wnt/*β*-catenin Signaling* pathways. Males activated signaling pathways related to *Neuregulin Signaling* consistent with upregulation of *Ereg, Areg, and Hbegf* genes (Figure 4). In addition, the activity of *HMGB1 Signaling* and *IL-17 Signaling* pathways additionally increased in males due to stronger upregulation of *IL-6, LIF, Fos, Fosl1*, and related genes. *GDNF Family Ligand Receptor Interactions, CSDE1 Signaling, Mouse Embryonic Stem Cell Pluripotency*, and *Aryl Hydrocarbon Receptor Signaling* demonstrated a male-specific up-regulation. *Cardiac Hypertrophy Signaling* and *Wound Healing* pathways were activated in males but inhibited in females.

Baseline expression levels of DEGs related to *Axonal Guidance Signaling* in both sexes due to a robust expression of endothelin-converting enzyme *Ecel1, Shh, Ngfr, Glis1*, and other related genes (Figure 4). *Cholesterol Biosynthesis, Reelin Signaling in Neurons, Synaptogenesis Signaling Pathway*, and *GP6 Signaling* pathways were downregulated in females but were not significantly altered in males. *Signaling by Rho Family GTPases, cAMP-mediated signaling*, and sulfated glycosaminoglycan biosynthesis were downregulated in both sexes. The iodothyronine deiodinase genes *Dio2* and *Dio2*, which regulate thyroid hormone conversion, increased in both sexes, yet the increase was higher in males. It is important to note that many DEGs related to pathways activated in males exhibited robust expression in females in both axotomized and sham nerves (Figure 4). Thus, male-specific post-injury DEG regulation balanced the activity of respective pathways to levels observed in females. The most significant sex-specific DEGs were further highlighted on heatmap diagrams grouped by gene relevance to peripheral nerve injury processes according to IPA predictions and GO terms.

### Estrogens as sex-dependent regulators of injury response

**FIGURE 5:**
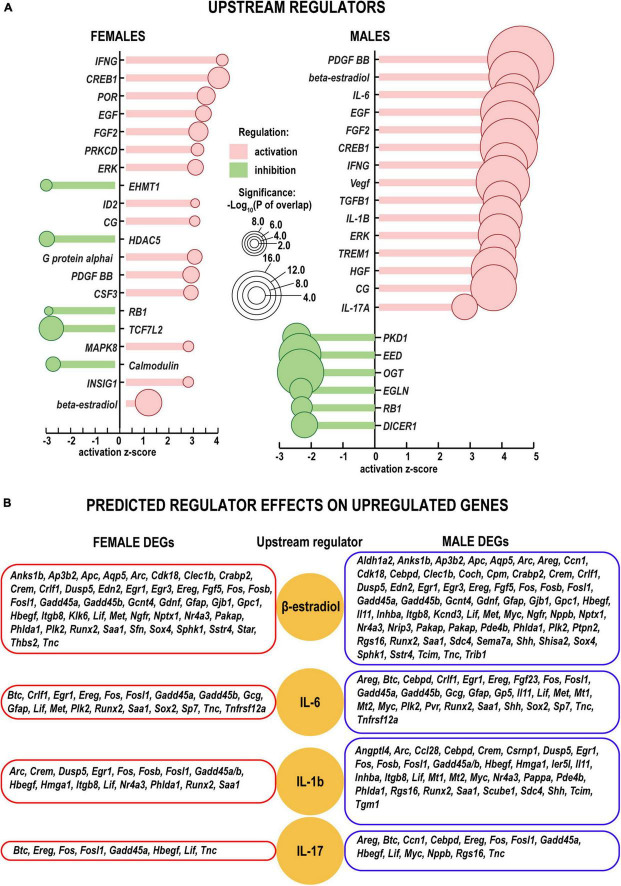
Upstream regulators of the injury response in distal nerve segments. **(A)** Activation (positive z, red color) and inhibition (negative z, green color) z-scores indicate IPA-predicted regulator effects to upregulate or downregulate respective DEGs. Circle diameters correspond to the *p*–values [as –log_10_(P) according to a significance scale] that measure the statistical significance of the overlap between the dataset DEGs and all genes regulated by a given regulator calculated using Fisher’s exact test in IPA. **(B)** Upregulation of DEGs in females (left panels) and males (right panels) (log_2_FC > 1, P_adj_ < 0.1). Yellow circles correspond to select upstream regulators (beta-estradiol, IL-6, IL-1b, and IL-17) predicted by IPA. All predicted upstream regulators are included in Supplementary Table 2.

**FIGURE 6:**
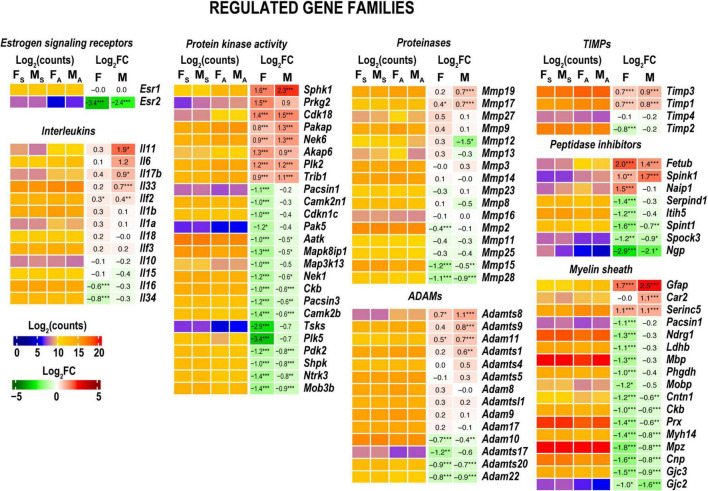
Regulated gene families. Heatmaps on panels display normalized log_2_(counts) in sample groups (F_S_, female sham; M_S_, male sham; F_A_, female axotomy; M_A_, male axotomy) and log_2_FC in female (F) and male (M); [*n* = 6 mice/group, 2 mice/sample (pooled), 3 sample/group] of respective DEGs. Log_2_FC significance was determine in DESeq2 using Wald test: *p ≤ 0.05; **p ≤ 0.005; ***p ≤ 0.0005. DEGs were sorted by log_2_FC.

The cascade of predicted upstream transcriptional regulators (small endogenous molecules, proteins, including transcription factors, and RNAs) could explain the observed gene expression changes. According to IPA predictions, growth factors (including *Egf, Fgf2, Vegf, Hgf*, and others) could contribute to transcriptional regulation in both sexes (Figure 5A). In addition, IPA predicted that steroid hormone β-estradiol could regulate downstream transcription distinctly in females in males. In females, among 144 DEGs targeted by β-estradiol, 44 DEGs were upregulated, and 68 DEGs were downregulated. In males, among 77 genes 64 were upregulated more than twofold but only 12 DEGs were downregulated (P_*adj*_ < 0.1) (Figure 5B and Supplementary Table 2). The *Esr1* gene, encoding one of two main types of nuclear estrogen receptors ERα that transmit β-estradiol signaling in the nucleus (Rettberg et al., 2014), maintained moderate levels of expression in both sexes. Remarkably, the *Esr2* gene encoding ERβ demonstrated a 10-fold decrease in females (Figure 6). In males, ERβ expression decreased fivefold, yet a moderate expression level was maintained. We proposed that estrogen receptor signaling could distinctly regulate early phase WD responses in nerves of both sexes, which is modulated, in part, via estrogen receptors’ sexually-dimorphic expression.

### Immune regulation in the distal stumps

Gene ontology (GO) analysis (Ashburner et al., 2000) identified a relatively small number of regulated DEGs generally attributed to the innate immunity system (GO term 0045087) (Figure 6). Likewise, IPA did not detect significant changes of immune-related canonical pathways (Figure 3). Consistent with time-lapse microarrays in axotomized male rats (Yu et al., 2016), robust baseline expression of pro-inflammatory interleukins IL-1b and IL-6 was observed in both sexes in axotomized and sham mice, albeit post-axotomy activation of IL-6 was stronger in males. Upstream regulator analysis in IPA predicted that interleukins IL-6 and IL-1B could promote upregulation of a larger number of DEGs in males, including important growth and transcription factors (Figure 5B and Supplementary Table 2).

### Sexual dimorphism of cellular phosphorylation

Both females and males attenuated expression levels of kinases, including protein kinases (Figure 6). Males predominantly increased *Sphk1* encoding sphingosine kinase 1 that catalyzes the phosphorylation of sphingosine, a lipid mediator with intra- and extracellular functions, including neuroinflammation. The following kinases showed a moderate mRNA increase. The protein kinase adapter gene involved in ubiquitin-dependent protein degradation *Trib1* (Satoh et al., 2013), *Plk2* (a serine/threonine-protein kinase involved in synaptic plasticity), cyclin-dependent kinase 18 (*Cdk18*), cGMP-dependent protein kinase 2 (*Prkg2*), paralemmin A kinase anchor protein (*Pakap*), and serine/threonine-protein kinase *Nek6* important for mitotic cell cycle progression. Females, but not males, exhibited significant downregulation of multiple kinases and kinase-associated protein genes, including *Cdkn1c, Plk3*, *Mapk3k13, Camk2n1*, and *Camk2b*.

### Regulation of (extra)-cellular proteolysis

In both sexes, MMPs gelatinase A (*Mmp2*), stromelysin-1 (*Mmp3*), neutrophil collagenase (*Mmp8*), gelatinase A (*Mmp9*), *Mmp19, Mmp23*, membrane-type MMPs (MT-MMPs) MT1-MMP (*Mmp14*), MT2-MMP (*Mmp15*), MT3-MMP (*Mmp16*), and MT4-MMP (*Mmp17*) genes exhibited high expression levels in both distal nerve stumps and sham at 24 h (Figure 6). Stromelysin-2 (*Mmp10*) exhibited a low expression level in both sexes. Several MMPs colocalized in the genomic locus on chromosome 9, Mmp12, Mmp13, and Mmp27, exhibited mild activation in females and a decrease in males. Many ADAM/ADAM-TS genes were expressed at high levels irrespective of sex (Supplementary Table 1). *Timp1*, *Timp2*, and *Timp3* but not *Timp4* expressed at high levels. *Timp1* and *Timp3* showed a mild increase in both sexes in axotomized nerves. MMP, ADAM/ADAM-TS, and TIMPs gene expression patterns were remarkably consistent in axotomized distal rat nerve stumps (Chernov et al., 2015), suggesting universal proteolytic activities.

### Myelin sheath remodeling

Both sexes reduced levels of many myelin-associated genes, including myelin basic protein (MBP), periaxin (*Prx*), myelin protein zero (*Mpz*), and myelin-associated glycoprotein (*Mag*). GFAP maintained high expression in females and was significantly increased in males to a matching mRNA level (Figure 6).

### Growth factor expression is sex-specific

Males, but not females, significantly upregulated *Neuregulin Signaling* implicated in growth factor-dependent repair and maintenance of the nervous system (Fricker and Bennett, 2011) (Figure 4). Rho GDP-dissociation inhibitor (RhoGDI) encoded by the *ARHGDIA* gene, p75 neurotrophin receptor (p75NTR, also named NGFR) demonstrated a twofold increase in both sexes. In males, we observed a 4–8-fold increase of the epiregulin (*Ereg*) and amphiregulin (*Areg*) autocrine growth factors, ligands of EGF receptor, known to stimulate Schwann cells in axotomized PNS (Rosenbaum et al., 1997; Meier et al., 1999; Jessen and Mirsky, 2021). The proheparin-binding EGF-like growth factor *Hbegf* associated with macrophage-mediated cellular proliferation exhibited a fourfold increase in males. In addition, probetacellulin (*Btc*) EGFR ligand showed eightfold upregulation in both sexes.

The *GDNF Family Ligand Receptor Interactions* signaling is regulated by a spectrum of polypeptide growth factors, including the *GDNF* (Gordon, 2009). Regulation of *Mouse Embryonic Stem Cell Pluripotency* was male-dominant potentially due to the increase of LIF extrinsic pluripotency factor accompanied by the induction of the intrinsic transcription factors *Sox2* and *c-Myc.*

### Expression of sex chromosome-linked genes

**FIGURE 7:**
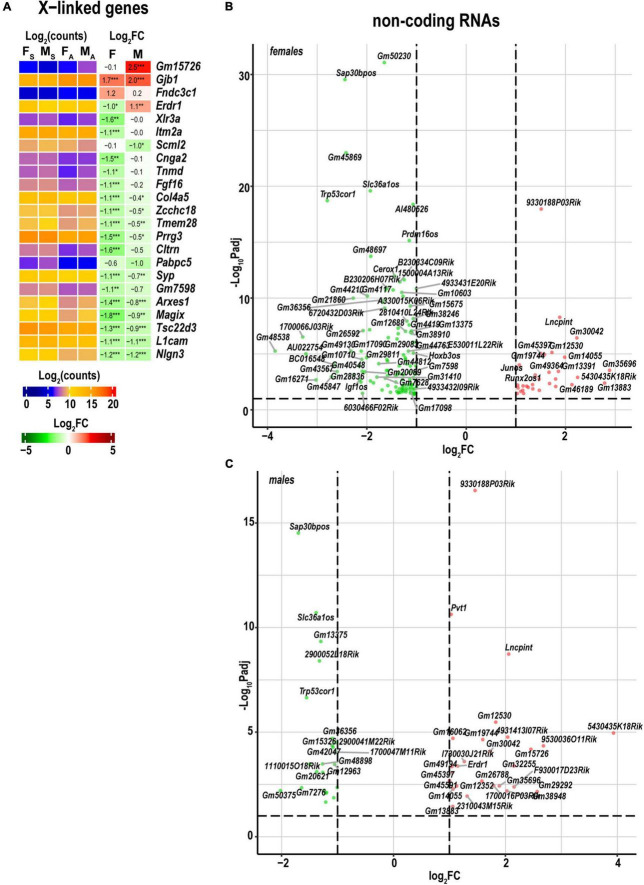
Regulated X-linked and ncRNA DEGs exhibited sexual dimorphism. **(A)** Regulated sex chromosome-linked DEGs. Heatmaps display normalized log_2_(counts) in sample groups (F_S_, female sham; M_S_, male sham; F_A_, female axotomy; M_A_, male axotomy) and log_2_FC in female (F) and male (M); [*n* = 6 mice/group, 2 mice/sample (pooled), 3 sample/group] of respective DEGs. Heatmap colors correspond to the respective scales. Log_2_FC significance was determine in DESeq2 using Wald test: *p ≤ 0.05; **p ≤ 0.005; ***p ≤ 0.0005. DEGs were sorted by log_2_FC. Significant ncRNA DEGs in female **(B)** and male **(C)** mice. Volcano scatter plots show –log_10_P_adj_ and log_2_FC. Red and green colors indicate up- and downregulated ncRNAs, respectively. Thresholds (log_2_FC > 1 or log_2_FC < –1) and –log_10_P_adj_ < 0.1 are shown by dotted lines. Selected DEGs are labeled by gene symbols.

As reported in the DRGs (Chernov and Shubayev, 2021) and proximal nerve stumps (Chernov and Shubayev, 2022) of the same animal cohort genomic localization of DEGs on a sex-chromosome could determine sexually dimorphic expression. Because females can up- or down-regulate the dosage of X-linked genes by female-exclusive X-chromosome inactivation epigenetic mechanism, the expression of X-linked genes could exhibit significant sexual dimorphism. In the distal stump, females upregulated two X-linked genes for the gap-junction β-1 protein (Gjb1) and the fibronectin type III domain-containing protein 3C1 gene (Fndc3c1). Gjb1 was also activated in males (Figure 7A). In addition, females reduced the expression of over a dozen genes, including Gm7598, Cltrn, Prrg3, Tmem28, Zcchc18, Col4a5, Fgf16, Tnmd, Cnga2, Itm2a, and Xlr3a that did not significantly change in males. Several Y-linked genes in males were strongly expressed, including lysine (K)-specific demethylase 5D (*Kdm5d)*, eukaryotic translation initiation factor Eif2s3y, DEAD box helicase Ddx3y, and ncRNAs (*Gm21860* and *Gm47283*) in both shams and axotomized nerves.

### ncRNAs differentially expressed genes exhibit female-dominant regulation

In the distal nerve stump, 172 ncRNA DEGs were detected, including 153 in females, and 51 in males (Figures 7B,C). Reduced expression of opposite strand ncRNA genes occurred in females and included *Hoxb3os, H3f3aos, Prdm16os, Rapgef3os2, Tspan32os, Tbx3os1, Pard3bos3, Slc36a1os, Igf1os*, and *Sap30bpos*. *Runx2os1*, and *Junos* genes exhibited upregulation in females. *Slc36a1os* and *Igf1os* genes were reduced in males.

### Crosstalk of cytokines and growth factors could regulate reprogramming in Wallerian degeneration

**FIGURE 8:**
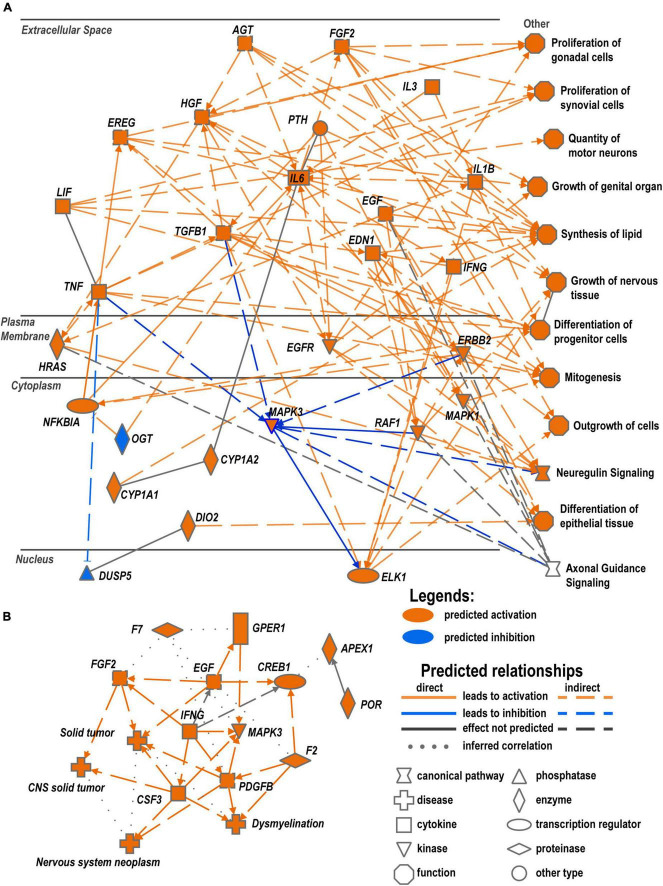
Crosstalk of cytokines and growth factors could regulate reprogramming in WD. **(A)** Predicted regulation of male-specific processes based on RNA-seq analysis. **(B)** Predicted female-specific regulatory network. Networks represent comprehensive synopsis that summarizes the most significant findings in the canonical pathway, upstream regulators, and disease and biological function categories of analysis. Diagrams were constructed in IPA using Graphical Summary function to visualize related biological activities.

Interactive network predictions in IPA suggested that networks of cytokines and growth factors, including neuron-specific growth factors, act via their specific receptors to positively regulate neuron growth and proliferation of cells of supporting tissues. The upregulation of these networks was higher in males as compared to females (Figure 8A). Cytokines localized in the extracellular milieu could interact with plasma membrane-associated EGFR, ERBB, and HRAS GTPase and mediation of cytoplasmic and nuclear factors. This regulatory network leads to the activation of axonal guidance, motor neuron outgrowth, mitogenic activity, lipids synthesis, and other pathways promoting axon regrowth. In addition, female-specific networks predicted signaling processes and metabolic changes that could cause a deficit in the myelin maintenance system, leading to pathologic states, such as dysmyelination (Figure 8B).

## Discussion

Sciatic nerve axotomy triggers a strong sex-specific early phase transcriptional response in regenerating (proximal) nerve stump (Chernov and Shubayev, 2022) and corresponding DRGs (Chernov and Shubayev, 2021). The present study documents sexual dimorphism in early phase WD response in the distal sciatic nerve segment of the same animal cohort.

WD is a prerequisite to a successful peripheral nerve repair post-injury (Gerdts et al., 2016) orchestrated by denervated Schwann cells. Due to their own remarkable plasticity, Schwann cells de-differentiate immediately after injury to acquire an injury-specific phenotype that drives a sequence of reparative processes in both neurons and immune cells (Arthur-Farraj et al., 2012; Jessen et al., 2015). Through a coordinated transcriptional reprogramming, Schwann cells expedite axonal and myelin proteolytic fragmentation, phagocytosis, and secretion of cytokines and chemokines to foster the immune cell recruitment (Tofaris et al., 2002; Chernov et al., 2015; Jessen et al., 2015).

Interleukins IL6 and IL1b could play a significant role to promote transcriptional reprogramming of a large number of genes in WD nerves, especially in males. Except for the moderately upregulated IL-17b, the panel-wide activation of the IL-17 family cytokines was not observed in either sex, consistent with the well-established notion that recruitment of IL-17-expressing immune [T helper (Th)17] cells starts days post-injury (Kim and Moalem-Taylor, 2011). It is important to note, that the upregulation of many cytokine genes in males led to the equalized absolute expression of respective mRNAs to matching levels in both sexes.

Estrogens, including β-estradiol, and targeted genes showed sex-dependent WD response. Both *Esr1* and *Esr2* are expressed in nerves of both sexes and the corresponding DRGs (Chernov and Shubayev, 2022). Injury induced a 10- and 5-fold decrease in *Esr2* in *f*emale and male nerves, respectively. Under acute pathological conditions, estrogens regulate the neuronal (Hucho et al., 2006) and immune functions (Straub, 2007). In the corresponding DRGs, *Esr1* signaling mediates the female-specific pain hypersensitivity (Khomula et al., 2017; Chernov et al., 2020). In the nervous system, rapid attenuation of estrogens in DRG can be complemented by a neuron-specific β-estradiol biosynthesis (Evrard and Balthazart, 2004). In addition to estrogens of gonadal origin (Rettberg et al., 2014) the downstream signaling is likely influenced by the naturally higher β-estradiol levels in females. We concluded that estrogen signaling is a potent regulator of early phase WD response in nerves of both sexes, which is modulated, in part, via estrogen receptors’ sexually-dimorphic expression.

Sex specificity in Schwann cell signaling, the dominant cell type in the damaged nerve within 24 h post-injury, manifested in myelin protein and growth factor expression patterns. Thus, the GDNF family of neurotrophic factors known as survival factors for neurons, bind to respective receptors, including upregulated NGFR, which could trigger the phosphorylation of RET tyrosine kinase receptor (Durbec et al., 1996) that is implicated in the regulation of PI3k/Akt and Plcγ/Ip3r dependent Ca^2+^ signaling during neurogenesis (Fukuda et al., 2002; Lundgren et al., 2012) and, sometimes, hyperalgesia (Bogen et al., 2008). In addition, GDNF/Gfra1 could interact with neural cell adhesion molecules such as NCAM to induce an axonal expansion (Nielsen et al., 2009). GDNF family of ligands can act in a synergetic manner with other growth factors, including transforming growth factor-β (Tgf-β) and sonic hedgehog (Shh) (reviewed in Jessen and Mirsky, 2021). GDNF signaling can stimulate the migration of neuronal precursors and Schwann cells (Iwase et al., 2005) to the site of the injury in both sexes. Action by the neurotrophin growth factors, their receptors, and RhoGDI could inhibit the Rho signaling pathway in both sexes and stimulate the neurite outgrowth (Yamashita and Tohyama, 2003; Dovas and Couchman, 2005).

Proteinases and their intrinsic inhibitors are indispensable for remodeling ECM after nerve damage, establishing a permissive environment for axonal regrowth, promoting regeneration and remyelination (Shubayev and Strongin, 2018; Pellegatta and Taveggia, 2019). MMP and disintegrin ADAM/ADAM-TS protease families control cytokine and growth factor receptors and their ligands in peripheral nerves, including IGF and neuregulin/ERBB after the sciatic nerve axotomy (English et al., 2000; Chattopadhyay and Shubayev, 2009; Liu et al., 2010). MMPs in the orthologous human chromosome 11q22.3 region could carry distinct histone modification marks (Chernov et al., 2010), epigenetic control of this set of metzincins could be involved. Consistent with our previous report (Chernov et al., 2015), of the ECM-remodeling metzincins, demonstrate regulated expression in injured nerves. Protease expression levels were high in intact and axotomized nerves. Processes of post-translational protease activation and binding to intrinsic proteinase inhibitors control potentially cytotoxic proteolytic activities. Aberrant cleavage of mediators of nociceptive signaling during neurogenesis could cause neuropathic pain (Remacle et al., 2015). TIMP family members are important regulators of extracellular proteolysis in normal and damaged nerves (Kim et al., 2012; Liu et al., 2015; Nishihara et al., 2015; Chernov and Shubayev, 2021). Timp1 and Timp3 demonstrated high levels of expression and further increased post-axotomy in both sexes.

Females reduced the expression of over a dozen of sex chromosome-related genes, including *Gm7598, Cltrn, Prrg3, Tmem28, Zcchc18, Col4a5, Fgf16, Tnmd, Cnga2, Itm2a*, and *Xlr3a* that did not significantly change in males. As reported in the DRGs (Chernov and Shubayev, 2021) and proximal nerve stumps (Chernov and Shubayev, 2022), at least in part, the sexual dimorphism may be attributed to differences in baseline expression of sex chromosome-linked genes rather than due to post-axotomy regulation.

Sexually dimorphic regulation of lipocalins of the major urinary protein (MUP) family was observed in our study. The male-prevalent activity of MUPs has been attributed to regulating metabolic rate, toxin removal, and survival (Penn et al., 2022). In addition, MUPs carry on chemical communication and pheromone signaling functions (Beynon and Hurst, 2003) in response to a physical injury.

The thyroid hormones (T4 and T3) play an essential role in peripheral nerve regeneration producing a lasting and stimulatory action on axotomized neurons and Schwann cells (Barakat-Walter, 1999). Limited data exist on their role in the development of demyelinating diseases (reviewed in Zorrilla Veloz et al., 2022). Disorders of thyroid metabolism are possible causes of inflammation and autoimmune reactions. Selenocysteine-containing Dio2 and Dio3 deiodinases are primarily responsible for T3/T4 enzymatic activation and inactivation, respectively. At 24 h post-axotomy, the upregulation of both enzymes was stronger in males. Thyroid hormone metabolic enzymes exhibited sexually dimorphic expression at the site of nerve axotomy. Their role in nerve injury response requires a focused investigation.

We predicted regulatory changes in female and male mice to constitute an immediate pro-regenerative response to nerve injury relative to shams. It is conceivable that, at least partially, sex differences in gene transcription could be matched over a more extended time course of nerve injury response. It is important to note that protein-level expression profiles cannot be directly interpreted using RNA-seq data. Single-cell RNA-seq, spatial transcriptomics analysis could provide additional functional information and a precise understanding of sexually dimorphic mechanisms.

## Materials and methods

### Reagents

Reagents and resources are listed in Supplementary Table 3.

### Animals

Female and male C57BL6/J mice (6–8 weeks old, Jackson Labs) were randomly assigned to axotomy (*n* = 6/group) and sham (*n* = 6/group). The mice were housed in a temperature-controlled room (∼22°C), on a 12 h light/dark cycle, and had free access to food and water. All procedures were conducted between 8.00 and 12.00 daytime. Under isoflurane anesthesia, the left sciatic nerve was exposed at the mid-thigh level. The entire width of the nerve was axotomized using sterile microsurgery scissors. In a sham surgery animal cohort, nerves were exposed using surgical scissors without transection. The muscle was then sutured, and the skin stapled. At 24 h after surgery, distal stumps of the axotomized sciatic nerve were collected for RNA isolation.

### Tissue samples

All surgical and tissue harvesting instruments were sterilized and repeatedly treated with RNase Away reagent followed by a rinse in RNase-free water. Tissues were submerged in 500 μl RNAlater stabilization solution, placed at 4°C overnight, then transferred for storage at −20°C. All sample groups were processed in parallel to minimize batch effects.

### RNA purification

DRG tissues from 2 animals were pooled to obtain at least 500 ng of total RNAs. Tissues were transferred in Trizol solution and disrupted by mechanical homogenization. Total RNAs were purified using RNeasy RNA purification reagents. RNA concentrations and quality were determined using NanoDrop absorbance ratios at 260/280 nm and 260/230 nm. RNA integrity was determined using the Agilent Bioanalyzer Nano RNA chip. Five hundred nanogram of total RNA samples with RIN ≥ 7.0 were used for RNA-seq.

### RNA-sequencing

mRNA libraries were generated following the TruSeq Stranded mRNA library preparation protocol (Illumina). In brief, the Poly-A enriched mRNAs were purified using poly-T oligo coupled magnetic beads, followed by mRNA fragmentation, first and second strands synthesis, cleaning on AMPure XP beads, and 3′-adenylation. Ligation of TruSeq dual-index adapters was used for barcoding. The quality of RNA-seq libraries was validated using qPCR. Libraries were sized on Agilent Bioanalyzer DNA high sensitivity chip and normalized. RNA-seq was performed using the paired-end 100 cycle program on the NovaSeq 6000 system at the Genomics High Throughput Facility (University of California Irvine). Base calls were recorded and converted to FASTQ files containing sequencing reads and the corresponding quality scores using Illumina software. Sequencing was conducted until we acquired at least 50 million paired-end reads per sample.

### Data alignment and preparation for differentially expressed gene analysis

Data analysis steps are summarized in Supplementary Figure 1. FASTQ files were processed using Ubuntu server 20.04 LTS (64-bit ARM). FASTQ files were filtered to remove low-quality bases, TruSeq dual-index adapter sequences, and unpaired reads using *Trimmomatic* (Bolger et al., 2014). Transcript-level quantification was performed using Salmon (Patro et al., 2017) in quasi-mapping mode using version M27 of the Gencode mouse genome. To correct systematic biases commonly present in RNA-seq data, both *-seqBias* and *-gcBias* features were used. Transcript- to gene-level conversion was done using *Tximeta* (Love et al., 2020). RNA-seq quantification data quality was assessed using *MultiQC* (Ewels et al., 2016).

### RNA-sequencing data analysis

Gene count matrices were imported into the *DESeq2* package (Love et al., 2014). Outliers were identified by Cook’s distance method and excluded. Dataset’s normalization was conducted using trimmed *M*-values (TMM). Log_2_FC we calculated using the Wald test. The adjusted (shrunken) log_2_FC values were calculated using the adaptive *t*-prior *apeglm* method (Love et al., 2014). Significant DEGs defined by P_adj_ < 0.1 and used in downstream analyses. Batch effects were controlled using *removeBatchEffect* (Ritchie et al., 2015) and *RUVseq* (Risso et al., 2014) functions. DEGs were visualized using *PCAtools*, *ComplexHeatmap*, and *EnhancedVolcano* R packages.

### System biology analysis

Biological interpretation of the regulated signaling pathways in female and male animals was conducted by the Ingenuity Pathway Analysis (IPA) as described previously (Chernov and Shubayev, 2021, 2022) based on causal network approaches (Krämer et al., 2014). Cut-off filtering was applied (Padj < 0.1, |Log2FC| > 1) for IPA. Signaling pathway regulation directionality and upstream regulator analysis were based on statistical z-scores calculated in IPA using default parameters. Ontology terms were retrieved from the GO public database (Ashburner et al., 2000).

## Data availability statement

The original contributions presented in this study are publicly available. This data can be found here: www.ncbi.nlm.nih.gov/geo/ (GSE182751 and GSE182709).

## Ethics statement

The animal study was reviewed and approved by the Institutional Animal Care and Use Committee at the VA San Diego Healthcare System.

## Author contributions

AC: conceptualization, methodology, software, formal analysis, investigation, data curation, writing—original draft, review and editing, and visualization. VS: conceptualization, resources, writing—review and editing, project administration, and funding acquisition. Both authors contributed to the article and approved the submitted version.

## Abbreviations

DEG: differentially expressed gene
DRG: dorsal root ganglia
ECM: extracellular matrix
ERBB/EGFR: epidermal growth factor receptor
ERK5/MAPK7: mitogen-activated protein kinase 7
ESR/ER: estrogen receptor
FC: fold change
IL: interleukin
IPA: Ingenuity Pathway Analysis
MMP: matrix metalloproteinase
ncRNA: non-coding RNAs
P_*adj*_: adjusted *P*-value
PNS: peripheral nervous system
RhoGDI: Rho GDP-dissociation inhibitor
RNA-seq: RNA sequencing
WD: Wallerian degeneration.
