# Supplementary material for: Sexual dimorphism of early transcriptional reprogramming in degenerating peripheral nerves
Source: Front Mol Neurosci. 2022 Oct 27;15:1029278. doi: 10.3389/fnmol.2022.1029278 (PMC9648404; doi:10.3389/fnmol.2022.1029278)
Supplement: Supplementary file 3 [file Table_3.pdf]

Supplementary Table 3. Key resources description.

Resource	Description	Source	Identifier	URL
Animals	Mice	Jackson Labs, Sacramento, CA	strain C57BL/6	
Disposables	BioMasher microhomogenizer	TaKaRa	9791B	
Disposables	QIAshredder	Qiagen, Carlsbad, CA	79656	
Reagent	Trizol reagent	Thermo Fisher Scientific, Carlsbad, CA	15596026	
Reagent	RNAlater Stabilization Solution	ThermoFisher Scientific, Carlsbad, CA	AM7020	
Reagent	RNAeasy Mini Kit	Qiagen, Carlsbad, CA	74104	
Equipment	Centrifuge	Eppendorf	5425	
Equipment	Tapestation	Agilent Technologies, San Diego, CA, USA	4200	
Equipment	Novaseq	(Illumina Inc., San Diego, CA, USA)	6000	
Software	Trimmomatic (settings: trimmomatic PE -phred33 ILLUMINACLIP:TruSeq3-PE.fa:2:30:10 LEADING:3 TRAILING:3 SLIDINGWINDOW:4:15 MINLEN:36)	(1)	Ver. 0.39	http://www.usadellab.org/cms/?page=trimmomatic
Software	Salmon	(2)	Ver. 1.9.0	https://salmon.readthedocs.io
Software	Tximeta	(3)	Ver. 3.11	https://www.bioconductor.org/packages/release/bioc/html/tximeta.html
Software	MultiQC	Stockholm University, Stockholm, Sweden (4)	Ver. 1.9	https://multiqc.info/docs/

Software	DESeq2	(5)	Version 1.36.0	https://www.bioconductor.org/packages/devel/bioc/vignettes/DESeq2/inst/doc/DESeq2.html
Software	Ingenuity Pathway Analysis	Qiagen, Carlsbad, CA	Winter 2021	https://digitalinsights.qiagen.com
Database	Mouse genome		M29 (GRCm39)	https://www.gencodegenes.org/mouse

REFERENCES

1. Bolger, A. M., Lohse, M., and Usadel, B. (2014) Trimmomatic: a flexible trimmer for Illumina sequence data. *Bioinformatics* **30**, 2114-2120
2. Patro, R., Duggal, G., Love, M. I., Irizarry, R. A., and Kingsford, C. (2017) Salmon provides fast and bias-aware quantification of transcript expression. *Nat Methods* **14**, 417-419
3. Love, M. I., Soneson, C., Hickey, P. F., Johnson, L. K., Pierce, N. T., Shepherd, L., Morgan, M., and Patro, R. (2020) Tximeta: Reference sequence checksums for provenance identification in RNA-seq. *PLoS Comput Biol* **16**, e1007664
4. Ewels, P., Magnusson, M., Lundin, S., and Käller, M. (2016) MultiQC: summarize analysis results for multiple tools and samples in a single report. *Bioinformatics* **32**, 3047-3048
5. Love, M. I., Huber, W., and Anders, S. (2014) Moderated estimation of fold change and dispersion for RNA-seq data with DESeq2. *Genome Biol* **15**, 550
6. Brionne, A., Juanchich, A., and Hennequet-Antier, C. (2019) ViSEAGO: a Bioconductor package for clustering biological functions using Gene Ontology and semantic similarity. *BioData Mining* **12**, 16
