# Supplementary figures and images for: Sexual dimorphism of early transcriptional reprogramming in degenerating peripheral nerves
Source: Front Mol Neurosci. 2022 Oct 27;15:1029278. doi: 10.3389/fnmol.2022.1029278 (PMC9648404; doi:10.3389/fnmol.2022.1029278)

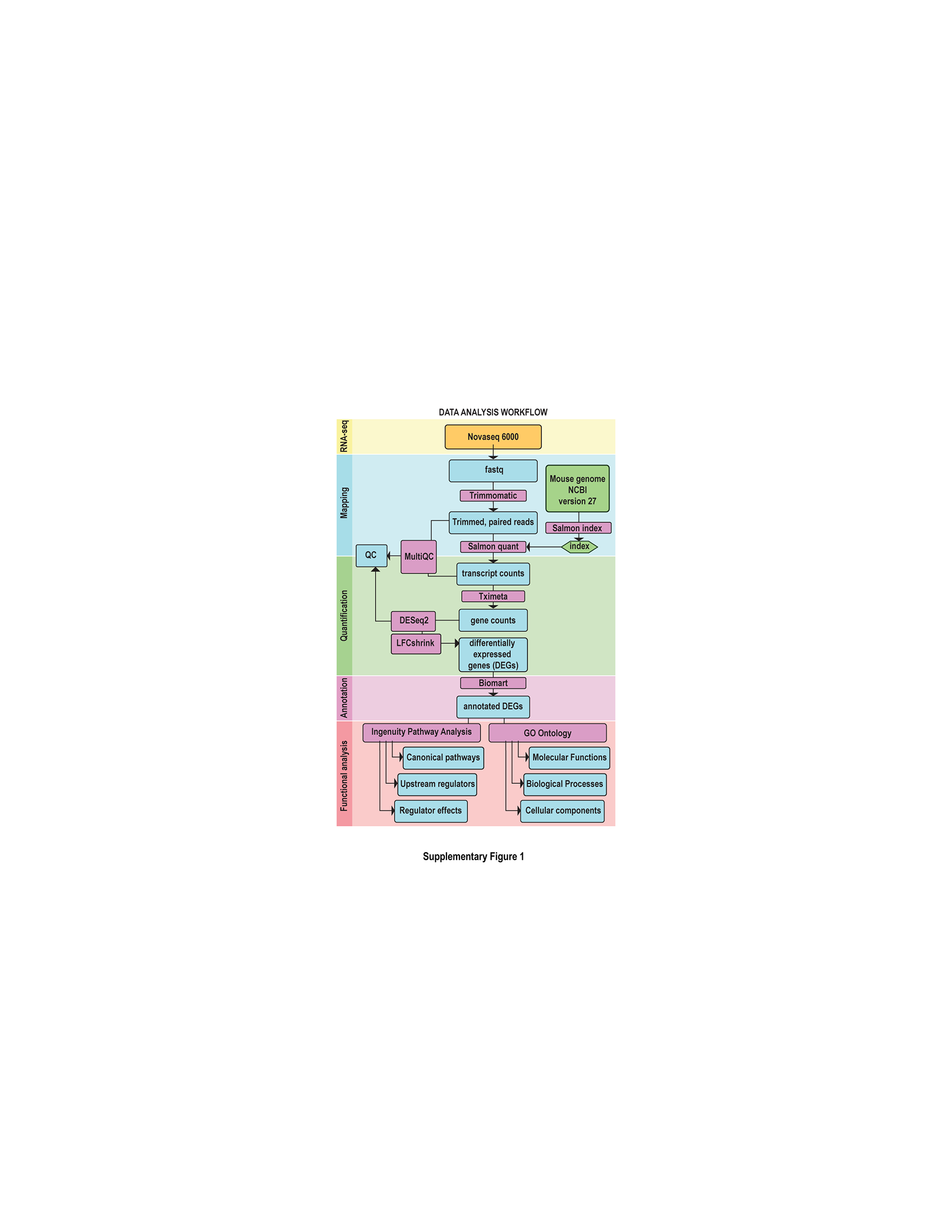

Supplement: Supplementary file 4 [file Image_1.TIF]
